# Expanding the Scope: Reflections on Digital Smoking Cessation Strategies for Diverse Age Groups

**DOI:** 10.2196/65929

**Published:** 2024-11-18

**Authors:** Bin Wei, Xin Hu, XiaoRong Wu

**Affiliations:** 1 The First Affiliated Hospital Jiangxi Medical College Nanchang University Nanchang, Jiangxi China

**Keywords:** digital smoking cessation, age group comparisons, behavioral health interventions, older adults, digital cessation treatment, cigarettes, tobacco, quit, telehealth, behavioral health, public health

We are writing to express our appreciation for the recent publication of the study entitled, “Expectations and Preferences for Digital Cessation Treatment: Multimethods Study Among Older Adults Who Smoke Cigarettes” in the *Journal of Medical Internet Research* [1]*.* I commend the authors for their efforts in exploring smoking cessation treatments within the older adult population and appreciate their focus on this often-overlooked demographic group. However, as I perused the article, I had a few concerns and suggestions that I would like to share with the authors and the editorial team to aid in the enhancement of future research.

First, the authors selected individuals aged 65 years and older as the focus of their study, specifically exploring their attitudes toward digital smoking cessation interventions. This choice is crucial, as older adults are at higher risk for tobacco-related health issues [2]. However, we believe that expanding the scope of the study to include younger populations, such as middle-aged adults, young adults, and adolescents, is equally important. Younger individuals also need effective smoking cessation tools, and their greater adaptability to digital technologies might allow them to benefit even more [3]. Future research could consider comparing the effectiveness of digital smoking cessation treatments across different age groups to gain a more comprehensive understanding of their applicability in various populations.

Moreover, for older adults with a long history of smoking, quitting can be an exceptionally challenging process. While digital smoking cessation tools offer convenience and accessibility, it remains to be seen whether these tools can effectively help older individuals overcome decades-long nicotine addiction. Future research might consider conducting long-term follow-ups to assess the sustained impact of digital cessation tools on older adults and explore whether they might benefit from additional support, such as in-person counseling or community-based programs, to enhance their chances of successfully quitting smoking.

Older adults in the process of quitting smoking require not only behavioral support but also emotional and social assistance. Whether digital smoking cessation tools can meet these more complex needs remains an open question. As a social worker, I believe that integrating more social and emotional support features into digital platforms, alongside traditional face-to-face counseling, could potentially offer a more comprehensive approach to smoking cessation support.

Overall, this article offers highly valuable insights into smoking cessation approaches for older adults, which can foster further exploration in the field of digital smoking cessation research. We would like to extend our gratitude once again to the authors for their efforts and to the editorial team for providing a platform to publish this important work.
